# Branched-Chain Amino Acids Supplementation Does Not Accelerate Recovery after a Change of Direction Sprinting Exercise Protocol

**DOI:** 10.3390/nu14204331

**Published:** 2022-10-16

**Authors:** Chutimon Khemtong, Antonio Tessitore, Salvador J. Jaime, Giuliana Gobbi, Jørgen Jensen, Ai-Lun Yang, Chia-Hua Kuo, Giancarlo Condello

**Affiliations:** 1 Institute of Sports Sciences, University of Taipei, Taipei City 111, Taiwan; 2 College of Sports Science and Technology, Mahidol University, Nakhon Pathom 73170, Thailand; 3 Department of Movement, Human and Health Sciences, University of Rome Foro Italico, 00135 Rome, Italy; 4 Department of Exercise and Sport Science, University of Wisconsin-La Crosse, La Crosse, WI 54601, USA; 5 Department of Medicine and Surgery, University of Parma, 43126 Parma, Italy; 6 Department of Physical Performance, Norwegian School of Sport Sciences, 0806 Oslo, Norway

**Keywords:** fatigue, creatine kinase, interleukin-6, muscle function, arterial stiffness, muscle soreness, inflammation, muscle regeneration

## Abstract

BCAAs supplementation has been widely used for post-exercise recovery. However, no evidence is currently available to answer the question of whether BCAAs supplementation can attenuate muscle damage and ameliorate recovery after a bout of change of direction (COD) sprinting, which is an exercise motion frequently used during team sport actions. This study aimed to assess the effect of BCAAs supplementation on muscle damage markers, subjective muscle soreness, neuromuscular performance, and the vascular health of collegiate basketball players during a 72 h recovery period after a standardized COD protocol. Participants orally received either BCAAs (0.17 g/kg BCAAs + 0.17 g/kg glucose) or placebo (0.34 g/kg glucose) supplementation before and immediately after a COD exercise protocol in a randomized, crossover, double-blind, and placebo-controlled manner. Creatine kinase increased immediately after exercise and peaked at 24 h, muscle soreness remained elevated until 72 h, whilst arterial stiffness decreased after exercise for both supplemented conditions. A negligibly lower level of interleukin-6 was found in the BCAAs supplemented condition. In conclusion, the results of this study do not support the benefits of BCAAs supplementation on mitigating muscle damage and soreness, neuromuscular performance, and arterial stiffness after COD for basketball players.

## 1. Introduction

Change of direction (COD) is a frequent motion that occurs during basketball, football, and many other team sport actions to decelerate the body within a transient period of time and to quickly reaccelerate towards a new direction while running or sprinting [1,2]. A COD task demands two types of muscle contractions: Eccentric contraction to decelerate and concentric contraction to accelerate the body towards a new direction. In particular, the eccentric muscle contraction is considered to be crucial for the deceleration phase and its implementation is emphasized in COD testing and training transfer [3]. Moreover, eccentric contractions are generally known to induce muscle damage [4]. Exercise-induced muscle damage (EIMD) is acknowledged to be a consequence of unaccustomed exercise [5], causing the disruption of intracellular muscle structure, sarcolemma and extracellular matrix, prolonged impairment of muscle function, delayed-onset muscle soreness (DOMS), stiffness, and swelling [5,6,7,8], and acting as a trigger to the inflammatory response. EIMD is directly linked to the inflammatory response, which is defined by five macroscopic pathological phenomena (i.e., swelling, elevated tissue temperature, redness, intensive sensation of a noxious stimulus, and functional impairment) [9]. Furthermore, EIMD is also related to macro- and micro-circulation dysfunctions, such as slower oxygen kinetics and limited oxygen delivery to the muscles [10], acute increase in arterial stiffness [11], and athletic performance impairment [12]. Muscle inflammation contributes to muscle regeneration, which immediately demands nutritional carbon and nitrogen sources for cell proliferation [13,14] during post-exercise recovery. Amino acids and protein are the main sources of dietary nitrogen.

Branched-chain amino acids (BCAAs), consisting of three essential amino acids (leucine, isoleucine, and valine), are commonly used supplements to accelerate fatigue recovery for high-intensity endurance performance [15]. The effects of BCAAs on the post-exercise muscle recovery have been widely studied under various forms of exercise. Previous meta-analyses [16,17,18,19] have reported an ergogenic benefit of BCAAs supplementation on post-exercise recovery from muscle damage [18,19], subjective feeling of soreness, and fatigue [16,17]. However, this effect has not yet been reported for COD sprinting, which is an action frequently used during many team sports.

To fill this knowledge gap, this study aimed to assess the effects of BCAAs supplementation on muscle damage and soreness, neuromuscular performance, and vascular health during a 72 h recovery from an acute bout of COD sprinting exercise protocol in collegiate basketball players. We hypothesized that BCAAs supplementation would attenuate muscle damage, muscle soreness, performance impairment, and arterial stiffness after a COD sprinting exercise protocol.

## 2. Materials and Methods

### 2.1. Study Design

This study is part of a research project granted by the Ministry of Science and Technology (Taiwan) and it has been approved by the University of Taipei Institutional Reviewer Board (Taipei, Taiwan, reference number: IRB-2019-079), with all experimental procedures being conducted in accordance with the Declaration of Helsinki [20].

A randomized, crossover, double-blind, and placebo-controlled study design was conducted to assess the time course of recovery after the EIMD under a condition of BCAAs or placebo supplementation. Time-point measurements were scheduled before and after the COD sprinting exercise protocol, and during the time course of recovery at 24 h, 48 h, and 72 h post-exercise for muscle damage and inflammatory biomarkers, perceived muscle soreness, neuromuscular performance, and vascular health. Blood lactate and glucose concentrations, as well as the rating of perceived exertion, were evaluated during the entire COD sprinting exercise protocol.

### 2.2. Participants

A minimum sample size of 13 participants was determined from an a priori power analysis performed by G*Power (version 3.1.9.2, University of Dusseldorf, Dusseldorf, Germany), for an F test (repeated measures, one group, within-factor, five measurements), with a statistical power (1-β) of 0.95, a large effect size (0.4), and an overall level of significance of 0.05 [21].

Twenty-three male collegiate basketball players belonging to the same university team were evaluated for their eligibility to participate in this study, in accordance with the following inclusion criteria: (a) age 18–25 years; (b) an experience in basketball training and competition for at least 5 years; (c) no musculoskeletal injuries during the last six months; (d) no smoking; (e) no medicine use; and (f) absence of known cardiovascular, pulmonary, metabolic, bone, or joint diseases. Participants were interviewed one month before the commencement of the study to confirm their fitness and health status, to ascertain the inclusion criteria, and to receive the explanation of all experimental procedures. They were asked to refrain from any supplementation use one month before the start of the first trial, to avoid exercise for three days before the first experimental session of each trial and during the time course of recovery (up to 72 h), and to abstain from alcohol and caffeine consumption during the 12 h prior to each experimental session.

Participants who satisfied all the inclusion criteria and agreed to follow all the instructions provided their written informed consent and were included in the final sample. Four players were excluded for the following reasons: Smoking (*n* = 1), injury in the month preceding the experimental session (*n* = 1), incomplete experimental session (*n* = 2). Finally, 19 participants (age = 19.8 ± 1.3 years, body mass = 75.9 ± 7.7 kg, height = 182.5 ± 7.0 cm, body mass index = 22.8 ± 1.9 kg/m^2^) were included in the final sample. At the time of the experimental sessions, the university team was on a regular training schedule, but it was not involved in any official basketball competition.

### 2.3. Experimental Protocol

**Figure 1:**
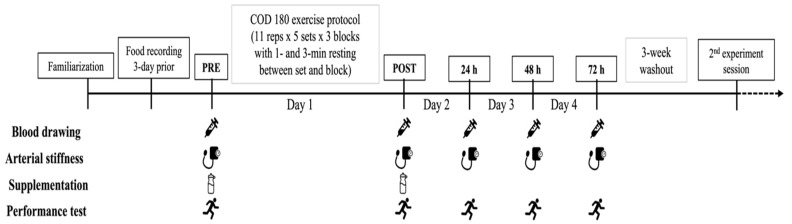
A schematic illustrating an overview of the experimental procedures of the present study.

Participants reported to the laboratory on nine occasions, one session for familiarization prior to the trials of four consecutive days for the experimental sessions, separated by a 3-week washout period (Figure 1). The first trial began one week after the familiarization session, with participants reporting to the laboratory in the morning at 8 am (±30 min) for the pre-exercise measurements (in the current order) related to vascular health, muscle damage and inflammatory biomarkers, and muscle soreness, followed by the ingestion of the first dose of supplementation (either BCAAs or placebo) at 30 min before the start of exercise protocol. Then, neuromuscular performance was evaluated just before the beginning of the exercise protocol. During the exercise protocol, blood lactate and glucose concentrations were measured, and the rating of perceived exertion (RPE) was asked to participants during (block 1-to-3) and after the exercise protocol. At the termination of the exercise protocol, post-exercise measurements included (in the current order) neuromuscular performance, vascular health, muscle damage biomarkers, and muscle soreness. Then, the rating of perceived exertion of the entire exercise protocol was provided by participants to represent their level of session RPE. The experimental session was completed with the ingestion of the second dose of supplementation (either BCAAs or placebo). Follow-up measurements were conducted during the time course of recovery at 24 h, 48 h, and 72 h post-exercise, including vascular health, muscle damage and inflammatory biomarkers, muscle soreness, and neuromuscular performance. After a 3-week washout period, all the experimental procedures were repeated under the other supplementation condition (either BCAAs or placebo).

Prior to the first experimental session of each trial, participants were required to record their diet for three days and they were asked to maintain their regular food intake for the duration of the four consecutive days of the experimental sessions. Moreover, two cans of Ensure (Abbott, Taipei, Taiwan) were provided to all participants to standardize the dietary contents and calories intake for dinner (the day before the first experimental session) and breakfast (2 h before the exercise protocol).

### 2.4. Exercise Protocol

Participants performed a COD sprinting exercise protocol consisting of 3 blocks of 5 sets of 11 consecutive 10 m sprints with 180° CODs (shuttle run), with a 3- and 1-min resting interval between block and set, respectively. The total distance covered during the exercise protocol was 1650 m with a total amount of 150 CODs. To guarantee maximal effort, strong verbal encouragement and time feedback were provided to participants during the exercise protocol. The total completion time of the exercise protocol (sum of block 1-to-3) and the completion time for each block (sum of set 1-to-5) were used for statistical analysis. The intensity of effort was evaluated with the measurement of blood lactate and glucose concentrations from a fingertip before, during (block 1-to-3), and after the exercise protocol using BIOSEN C-line (EKF-diagnostic, Barleben, Germany). Fingertip whole blood (20 μL) was drawn into an end-to-end capillary and mixed with 1 mL of hemolysis solution. Lactate and glucose concentrations were determined by the enzymatic-amperometric method. The chip sensor contained immobilized lactate and glucose oxidase catalyzing the oxidation of lactate to lactic acid and glucose to gluconic acid and hydrogen peroxide, which is oxidized at the electrode, thereby generating electrons. The results in the amperometric signal (sensor current) are proportional to the lactate concentration in the sample. Moreover, participants were asked to provide their rating of perceived exertion (RPE) after each block and 30 min after the end of the entire exercise protocol to represent the session RPE using the Borg CR-10 Scale [22].

### 2.5. Supplementation Strategy

In the current study, a pre- and post-exercise supplementation strategy was applied [23]. A total dosage of BCAAs (0.17 g/kg BCAAs + 0.17 g/kg isocaloric glucose) [24] or placebo (0.34 g/kg isocaloric glucose) supplement was split in two portions, mixed with 200 mL of water, and given to the participants at 30 min before and after the exercise protocol. The BCAAs supplement had a 4:1:1 ratio for leucine, isoleucine, and valine and an absorption time of 30 min, as certified by the provider (Ajinomoto Co., Inc., Chuo, Tokyo, Japan). A member of the research team was not directly involved during the experimental session but was responsible for the randomization of participants into the two crossover conditions and for the preparation of the BCAAs or placebo supplement for the first days of each trial.

### 2.6. Anthropometric Evaluation

Body mass (kg) and height (cm) were recorded to the nearest decimal using a Jenix DS-102 stadiometer (Dong Sahn Jenix Co., Ltd., Seoul, Korea). Participants were asked to wear the same and light sportwear, and to remove socks and all accessories. Then, participants stood on the measurement place of the stadiometer with normal breathing and no movement during the measurement. Two measurements were executed and averaged for statistical analysis.

### 2.7. Muscle Damage and Inflammatory Biomarkers

Venous whole blood samples (10 mL each) were collected from the basilica vein with 2 tubes of prechilled 5 mL evacuated tubes (BD Vacutainer Plus Plastic serum tubes, spray-coated silica) before (PRE) and after (POST, 24 h, 48 h, and 72 h) the exercise protocol. The blood samples were left at room temperature for 30 min to allow for clotting. Then the blood samples were centrifuged at 3000 rpm at 4 °C for 15 min to separate the serum from the whole blood contents. The serum was divided into Eppendorf tubes and stored at −80 °C in the refrigerator until the beginning of the analysis process. Creatine kinase (CK) and interleukin-6 (IL-6) were assessed as the indirect markers of muscle damage and the inflammatory mediator [25], respectively. The enzymatic analyzes for the CK concentration (CK-NAC; Beckman Coulter Inc., California) was performed in duplicate at each time point. For circulating IL-6 levels, the commercially available sandwich enzyme-linked immunosorbent assay (ELISA; High sensitivity Human IL6 Assay, Thermofisher, Waltham, MA, USA) was used for the analysis. The results in the plates were read in duplicate on the diagnostic automation at selected wavelengths (450 nm, with correction at 630 nm). The reported sensitivity of the ELISA was 0.03 pg/mL.

### 2.8. Muscle Soreness

Perceived muscle soreness was assessed before (PRE) and after (POST, 24 h, 48 h, and 72 h) the exercise protocol with the Visual Analog Scale (VAS) using a continuous 10 cm scale anchored by two verbal descriptors labeled from the left (no pain) to the right (worst possible pain) [26].

### 2.9. Neuromuscular Performance

The 505 COD test [1,27] was conducted on an indoor running track before (PRE) and after (POST, 24 h, 48 h, and 72 h) the exercise protocol. Since the test required the execution of a single turn and uncertainty might exist as to the leg preference and dominance in making directional changes, for each participant the fastest trial between the two legs was identified by performing 2 trials for each leg during the pre-exercise measurement. Subsequently, during the post-exercise and follow-up measurements, only 2 trials with the fastest leg were executed. According to standard procedures [27], participants had to sprint forward for 15 m, make a 180° COD, and come back to the starting line. Using a timing light system to the nearest 0.001 s (Smartspeed, Fusion Sport, Coopers Plains, Australia), the time to complete the initial 10 m linear sprint and the total 505 time was registered, while the COD deficit was calculated as the difference between the total 505 time and 10 m linear sprint. Each trial was separated by a 1 min resting period. If participants changed direction before the turning point, they were asked to complete another trial after the resting period. The fastest trial of the 505 COD test was used for statistical analysis.

### 2.10. Vascular Health

Vascular health was evaluated using the Omron VP-1000 vascular profiler (Colin Co. Ltd.; Komaki, Japan) before (PRE) and after (POST, 24 h, 48 h, and 72 h) the exercise protocol, considering the brachial-ankle pulse wave velocity (baPWV), ankle-brachial index (ABI), blood pressure parameters (brachial systolic blood pressure: brachial SBP, brachial diastolic blood pressure: brachial DBP, ankle systolic blood pressure: ankle SBP, and ankle diastolic blood pressure: ankle DBP) and brachial (brachial MAP) and ankle (ankle MAP) mean arterial pressure. Participants rested in the supine position with their eyes open and breathed normally for 2 min at a temperature of 23 ± 2 °C in a light controlled room. Four occlusive cuffs pressures were strapped on the right and left side of arm (about 1 inch over the foldable joint or elbow to represent the values from the brachial area) and ankle (about 1 inch over the anklebone to represent the values from ankle area). The measurement was performed two times and were automatically calculated using a machine [28]. ABI and baPWV values were averaged from the right and left sides and used for statistical analysis.

### 2.11. Statistical Analysis

Statistical analysis was conducted using the statistic software Statistical Package for the Social Sciences (IBM Corp., Version 23.0, Armonk, NY, USA). The level of statistical significance was set at *p* < 0.05. All the results are presented as mean ± SD. The normal distribution of the data was examined by a Shapiro–Wilk test, which confirmed the normal distribution of data for the majority of variables, except for CK and IL-6, which have been log-transformed (Log-10). Therefore, considering the specific variables and time-point measurements, the following statistical tests were applied: A paired *t*-test was applied to ascertain the differences between BCAAs and placebo supplementation for the time to complete the exercise protocol, energy intake, and session RPE. A two-way repeated measured ANOVA was applied to ascertain the effect of two within-subject factors, supplementation (BCAAs, placebo) and time (PRE, Block 1, Block 2, and Block 3, POST) on the dependent variable’s glucose and blood lactate. A two-way repeated measured ANOVA was applied to ascertain the effect of two within-subject factors, supplementation (BCAAs and placebo) and time (Block 1, Block 2, and Block 3) on the dependent variable RPE. A two-way repeated measured ANOVA was applied to ascertain the effect of two within-subject factors, supplementation (BCAAs and placebo) and time (PRE, POST, 24 h, 48 h, and 72 h) on the dependent variables, namely CK, IL-6, perceived muscle soreness, neuromuscular performance, and vascular health variables. Effect sizes for the main effects were calculated as the partial eta squared (*ηр*^2^) and interpreted as a small (0.01–0.06), medium (0.06 < *ηр*^2^ < 0.14), and large (> 0.14) effect. In the case of significant interactions, pairwise comparisons (*t*-test) with Bonferroni adjustments were applied. Cohen’s d was calculated for each comparison and was interpreted as a trivial (< 0.19), small (0.20–0.59), moderate (0.60–1.19), large (1.20–1.99), very large (2.0–4.0), and extremely large (>4.0) effect [29]. The associations between the variables were determined by Pearson’s product–moment correlations. Due to the original CK data, it was not normally distributed and Spearman’s Rho correlation was used to evaluate the associations among muscle damage, muscle soreness, and vascular health variables. The strength of associations was quantified and interpreted according to the following categories: ≤ 0.1 (trivial), 0.11–0.30 (small), 0.31–0.50 (moderate), 0.51–0.70 (large), 0.71–0.90 (very large), and ≥0.9 (nearly perfect) [30].

## 3. Results

The exercise protocol was performed with the same completion time under the two conditions, specifically, 505 ± 29 s for the BCAAs condition and 506 ± 29 s for the placebo condition. However, independently of the condition, the analysis of completion time for each block demonstrated significant differences. Block 1 (162 ± 7 s) was executed with a lower time, as compared to block 2 (170 ± 12 s, *p* < 0.001, d = 0.81) and block 3 (173 ± 14 s, *p* < 0.001, d = 0.99). No difference emerged for the calory intake recorded before the two trials (BCAAs: 1817 ± 310 kcal, placebo: 1812 ± 303 kcal).

### 3.1. Exercise Intensity

**Figure 2:**
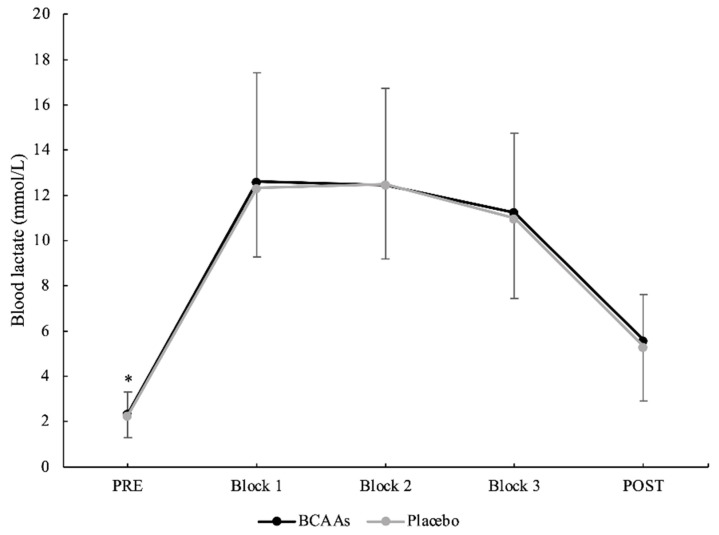
Blood lactate concentration during the exercise protocol. (*) Denotes differences (*p* < 0.05) from all time-point measurements.

A main effect of time without interaction was found for the blood lactate concentration (*p* < 0.001, *ηр*^2^ = 0.89) and RPE score (*p* < 0.001, *ηр*^2^ = 0.75). Compared to the baseline (2.30 ± 0.96 mmol/L), the blood lactate concentration was significantly higher in block 1 (12.47 ± 3.98 mmol/L, *p* < 0.001, d = 3.51), block 2 (12.47 ± 3.76 mmol/L, *p* < 0.001, d = 3.71), block 3 (11.12 ± 3.48 mmol/L, *p* < 0.001, d = 3.46), and at POST (5.44 ± 2.18 mmol/L, *p* < 0.001, d = 1.87) (Figure 2). Moreover, the blood lactate concentration was significantly elevated in all blocks of exercise, as compared to POST (block 1: *p* < 0.001, d = 2.19; block 2: *p* < 0.001, d = 2.29; and block 3: *p* < 0.001, d = 1.96, respectively). RPE was significantly higher in block 3 (8.3 ± 1.6 AU), as compared to block 1 (6.1 ± 2.1 AU, *p* < 0.001, d = 1.20) and block 2 (7.5 ± 1.6 AU, *p* < 0.001, d = 0.51). Furthermore, RPE was also significantly greater (*p* < 0.001, d = 0.75) in block 2, as compared to block 1. There were no differences in the session RPE between supplementation conditions (BCAAs = 7.9 ± 1.6 AU; placebo = 7.5 ± 1.3 AU).

### 3.2. Muscle Damage and Inflammatory Biomarkers

**Figure 3:**
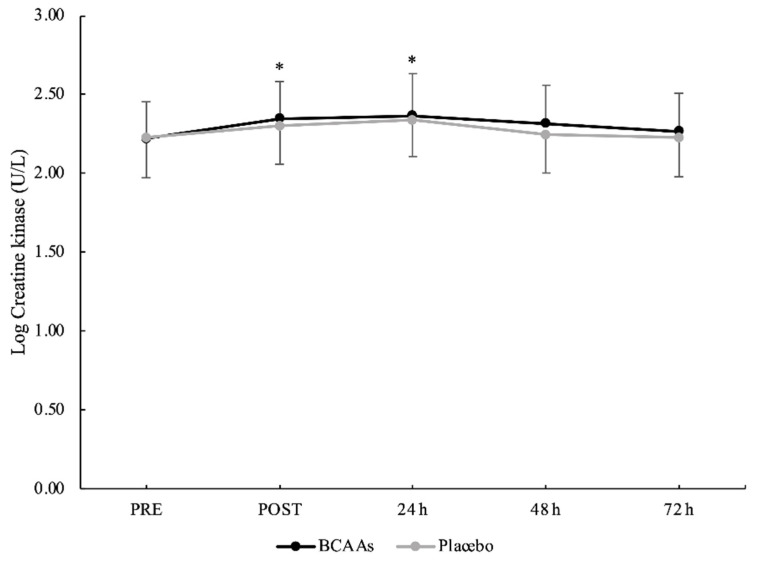
Creatine kinase serum activity with logarithmic transformation during the time course of recovery. (*) Denotes differences (*p* < 0.05) from PRE.

**Figure 4:**
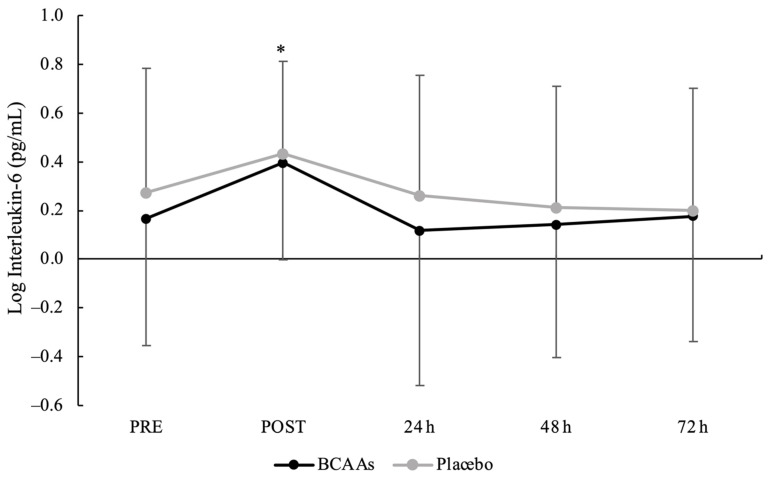
Interleukin-6 serum activity with logarithmic transformation during the time course of recovery. (*) Denotes differences (*p* < 0.05) from all time-point measurements.

A main effect of time without interaction emerged for CK (*p* = 0.014, *ηр*^2^ = 0.23). Compared to PRE (2.22 ± 0.24 U/L), CK was significantly elevated at POST (2.32 ± 0.24 U/L, *p* < 0.001, d = 0.41) and peaked at 24 h (2.35 ± 0.25 U/L, *p* < 0.001, d = 0.52) after exercise (Figure 3). Moreover, the CK at 24 h was also significantly higher than 48 h (2.28 ± 0.24 U/L, *p* < 0.001, d = 0.29) and 72 h (2.25 ± 0.24 U/L, *p* < 0.001, d = 0.43) after exercise. For IL-6 (Figure 4), both main effects of supplementation (*p* = 0.045, *ηр*^2^ = 0.21) and time (*p* < 0.001, *ηр*^2^ = 0.40) were found, without the interaction. For the supplementation effect, the BCAAs (0.199 ± 0.502 pg/mL) were significantly lower than the placebo (0.275 ± 0.468 pg/mL) condition. For the time effect, IL-6 peaked at POST (0.414 ± 0.382 pg/mL) and was significantly higher compared to PRE (0.219 ± 0.512 pg/mL, *p* = 0.001, d = 0.43), 24 h (0.189 ± 0.566 pg/mL, *p* = 0.002, d = 0.47), 48 h (0.178 ± 0.516 pg/mL, *p* < 0.001, d = 0.52), and 72 h (0.187 ± 0.503 pg/mL, *p* < 0.001, d = 0.51) after exercise.

### 3.3. Muscle Soreness

**Figure 5:**
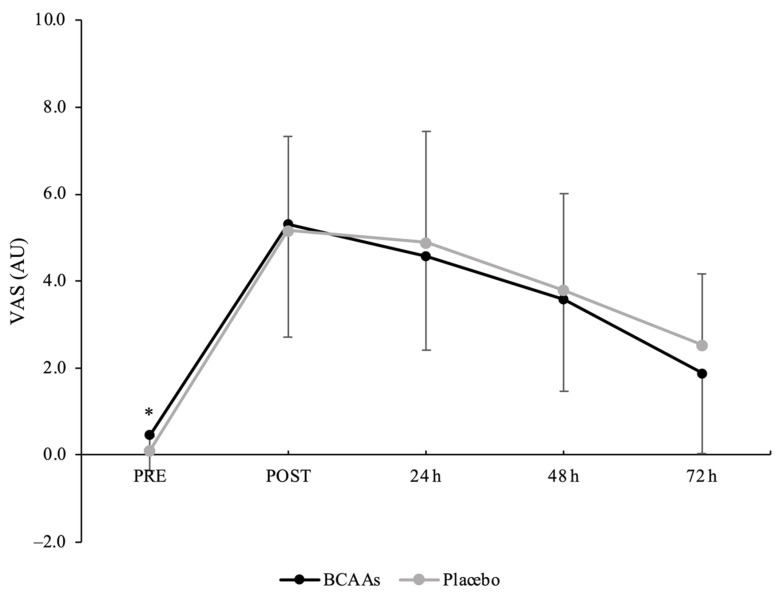
Muscle soreness scored by visual analog scale (VAS) during the time course of recovery. (*) Denotes differences (*p* < 0.05) from all time-point measurements.

A main effect of time without interaction was found for the VAS scale (*p* < 0.001, *ηр*^2^ = 0.74) (Figure 5). Compared to PRE (0.3 ± 0.7 AU), the perception of muscle soreness was significantly elevated at POST (5.2 ± 2.4 AU, *p* < 0.001, d = 2.85), 24 h (4.7 ± 2.3 AU, *p* < 0.001, d = 2.58), 48 h (3.7 ± 2.1 AU, *p* < 0.001, d = 2.14), and 72 h (2.2 ± 1.8 AU, *p* < 0.001, d = 1.45) after exercise. Moreover, the values at 48 h (*p* = 0.007, d = 0.69) and 72 h (*p* < 0.001, d = 1.45) were still elevated compared to POST.

### 3.4. Neuromuscular Performance

**Figure 6:**
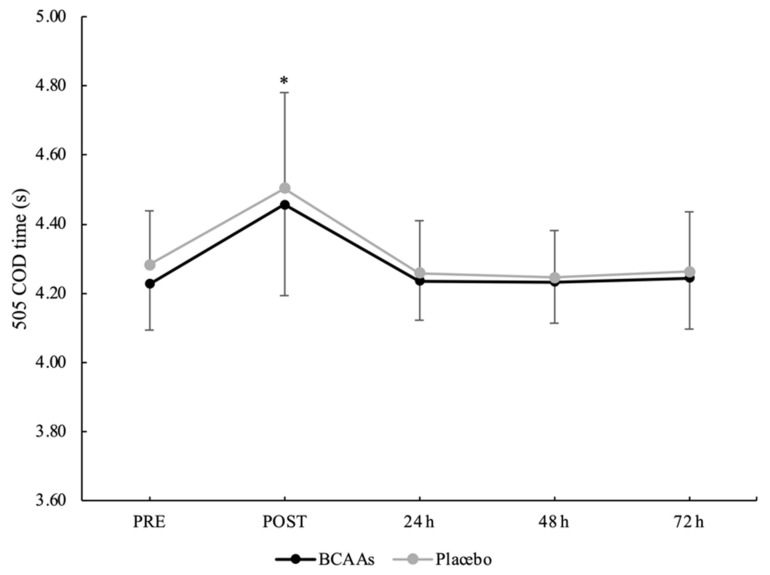
Total time for the 505 COD test during the time course of recovery. (*) Denotes differences (*p* < 0.05) from all time-point measurements.

**Figure 7:**
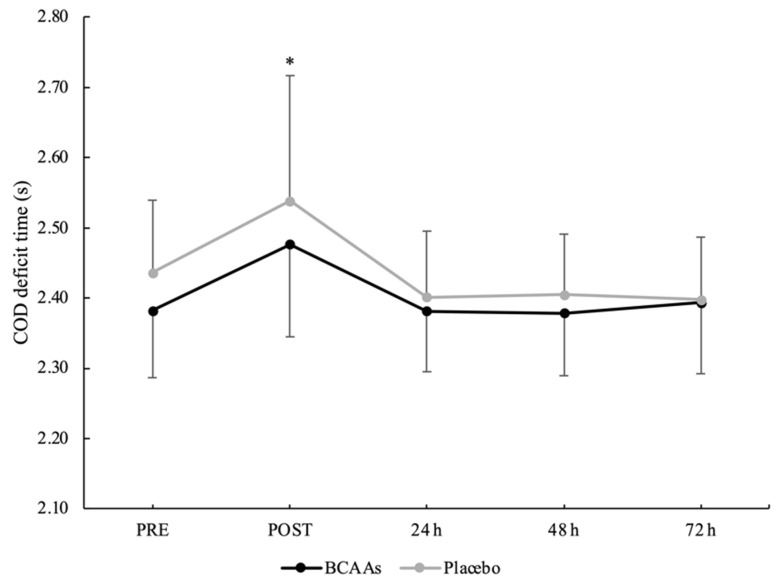
COD deficit time during the time course of recovery. (*) Denotes differences (*p* < 0.05) from all time-point measurements.

A main effect of time without interaction emerged for a total 505 COD time (*p* < 0.001, *ηр*^2^ = 0.60) (Figure 6). The total 505 COD time was significantly higher at POST (4.479 ± 0.267 s) compared to PRE (4.254 ± 0.146 s, *p* < 0.001, d = 1.05), 24 h (4.247 ± 0.132 s, *p* < 0.001, d = 1.10), 48 h (4.239 ± 0.125 s, *p* < 0.001, d = 1.15), and 72 h (4.235 ± 0.159 s, *p* < 0.001, d = 1.03) after exercise. For the COD deficit, a main effect of supplementation (*p* = 0.024, *ηр*^2^ = 0.25) and time (*p* < 0.001, *ηр*^2^ = 0.52) emerged without interaction. For the supplementation effect, COD deficit was significantly lower for the BCAAs (2.402 ± 0.079 s, *p* = 0.024, *ηр*^2^ = 0.25) compared to the placebo (2.436 ± 0.093 s) condition. For the time effect, post hoc analysis confirmed a higher COD deficit at POST (2.508 ± 0.158 s) compared to PRE (2.409 ± 0.102 s, *p* = 0.001, d = 0.74), 24 h (2.391 ± 0.090 s, *p* < 0.001, d = 0.91), 48 h (2.392 ± 0.087 s, *p* < 0.001, d = 0.91), and 72 h (2.396 ± 0.094 s, *p* < 0.001, d = 0.86) after exercise (Figure 7).

### 3.5. Vascular Health

Vascular health variables are shown in Table 1 for both BCAAs and the placebo condition. A main effect of time without interaction was found for ABI (*p* < 0.001, *ηр*^2^ = 0.70), baPWV (*p* = 0.011, *ηр*^2^ = 0.16), ankle SBP (*p* < 0.001, *ηр*^2^ = 0.65), and ankle DBP (*p* = 0.006, *ηр*^2^ = 0.142). ABI values were significantly lower at POST compared to PRE (*p* < 0.001, d = 1.76), 24 h (*p* < 0.001, d = 1.87), 48 h (*p* < 0.001, d = 1.89), and 72 h (*p* < 0.001, d = 2.00). baPWV values showed a significant decrease at POST (*p* = 0.011, d = 0.52), 24 h (*p* = 0.013, d = 0.42), and 48 h (*p* = 0.004, d = 0.46) after exercise compared to PRE. For the blood pressure parameters, ankle SBP was significantly lower at POST compared to PRE (*p* < 0.001, d = 1.09), 24 h (*p* < 0.001, d = 1.10), 48 h (*p* < 0.001, d = 1.01), and 72 h (*p* < 0.001, d = 1.29) after exercise. Moreover, the ankle SBP was also significantly lower at 48 h compared to 72 h (*p* = 0.032, d = 0.27). Ankle DBP significantly decreased at POST compared to PRE (*p* = 0.006, d = 0.43). There were no significant changes for brachial SBP, brachial DBP, brachial MAP, and ankle MAP at any time-point measurement.

### 3.6. Analysis of Associations

The potential explanation for the changes at 24 h after the exercise protocol was achieved through the investigation of the association of muscle damage with muscle soreness and arterial stiffness (Table 2). There was a negative moderate correlation between muscle soreness and baPWV (*r* = −0.477, *p* = 0.039). Moreover, the results from the Spearman correlation also revealed a large positive correlation between muscle damage (CK) and muscle soreness (VAS) (*r* = 0.694, *p* = 0.001). However, there was no correlation between CK and arterial stiffness variables at any time-point measurement. The results from the Pearson correlation indicated trivial to moderate associations between muscle soreness and arterial stiffness variables.

## 4. Discussion

The current study aims to investigate the effects of BCAAs supplementation on muscle damage and soreness, neuromuscular performance, and vascular health during the time course of recovery after a COD sprinting exercise protocol in collegiate basketball players. Contrary to our hypotheses, no beneficial effects of BCAAs supplementation were found on the markers for EIMD and vascular health. The main findings of this study are that (a) CK reached the peak at 24 h, returning to the baseline level at 48 h, (b) IL-6 peaked at post-exercise and returned immediately at the baseline level, (c) a higher IL-6 serum in placebo compared to the BCAAs condition, (d) the perception of muscle soreness did not return to the baseline level after 72 h, (e) the neuromuscular performance was impaired only at post-exercise, (f) the COD deficit might be a sensitive parameter to detect small changes due to an overall better response under the BCAAs supplementation condition, (g) arterial stiffness decreased immediately after exercise and remained low until 48 h after exercise. The current evidence can only be applied by considering the proposed supplementation strategy and the designed COD sprinting exercise protocol. Nonetheless, this study can advance the knowledge into the area of sport nutrition, which is the first to propose the application of BCAA supplementation and an EIMD characterized by repeated bouts of COD sprinting.

BCAAs supplementation did not influence CK activity. For both supplementation conditions, CK activity increased immediately after exercise and reached the peak at 24 h, suggesting that the proposed exercise protocol was able to induce a certain magnitude of muscle damage. The observed CK activity profile is in line with previous studies on eccentric resistance exercise [31] and drop jumps [32], showing the peak of CK release at 24 h post-exercise, even if CK could peak at different follow-ups after exercise [33,34]. A recent study, investigating a 90° COD exercise protocol [35], demonstrated the peak of CK activity immediately after the exercise. It could be speculated that the greater angle of the COD (180°) and the total number of CODs (150 repetitions) of the current exercise protocol induced a delayed and prolonged CK release.

IL-6 can act as a pro-inflammatory cytokine and an anti-inflammatory mediator for the regeneration process [36] by stimulating the proliferation and differentiation of myoblasts [37], and its production during exercise is related to the intensity and duration of exercise [25]. The results from the current study are consistent with previous studies [38,39,40] that showed a significant increment of IL-6 concentrations immediately after a prolonged running protocol, followed by a return at the baseline level. The increase of IL-6 after eccentric-based aerobic exercise could be explained by a greater elicit of hemodynamic changes that possibly contribute to increased spillover to the systemic circulation [41]. In the current study, the interaction supplementation x time did not emerge, probably due to the high variability in the data, but the main effect of supplementation highlighted a higher IL-6 release in the placebo condition compared to the BCAAs condition. A previous study did not demonstrate an effect of BCAAs supplementation in attenuating the IL-6 concentrations after resistance exercise in untrained males [33]. The potential underlying mechanism could be explained as the involvement of BCAAs supplementation in the activation process of inflammatory responses more than the attenuation of IL-6 release after EIMD [42]. BCAAs have the role of triggering the formation of reactive oxygen species in peripheral blood mononuclear cells, such as neutrophils, and lead to the expression of pro-inflammatory cytokines as IL-6 in the pathway of nuclear factor-kappa B activation [42]. Therefore, pre-exercise BCAAs supplementation could anticipate the activation of inflammatory response and induce a greater adaptive response, especially in the muscle regeneration process. In addition, it could also be speculated that the muscle regeneration process has been stimulated under the BCAAs condition compared to the placebo condition due to the availability of nitrogen sources from amino acids [13,14]. However, the current preliminary findings require further investigations to confirm the potential mechanisms of the effect of BCAAs supplementation on IL-6 concentrations after the COD sprinting exercise protocol in trained populations.

In the current study, the perception of muscle soreness peaked immediately post-exercise, gradually decreased afterwards, but it remained elevated until 72 h after exercise, even if the CK concentration already returned to the baseline level. While the elevation of both muscle soreness and CK are considered to be consequences of EIMD, they are not consistently associated. Indeed, a large positive association between muscle damage and soreness emerged only at 24 h post-exercise. The peak of muscle soreness after unaccustomed eccentric exercise can vary during the time course of recovery [43]. Furthermore, previous evidence demonstrated poor correlations between muscle soreness and other indirect markers of EIMD, raising the concern of whether muscle soreness clearly reflects the magnitude of muscle damage [43,44]. Possible explanations of underlying mechanisms of the muscle soreness after EIMD are structural damage within skeletal muscles rather than only through myofiber due to high mechanical stress loading on the load-bearing capacity of the sarcomere that leads to micro-ruptures inside or near Z-disk [45]. Moreover, muscle soreness might be related to the collagenous connective tissue, which is either located in musculotendinous junction or the extra-muscular fascia [46]. The collagenous connective tissue will act as a shock absorber to take up the excessive, eccentric loading that might induce muscle damage. 

The current study did not demonstrate beneficial effects of BCAAs supplementation on muscle damage and soreness alleviation by applying a pre- and post-exercise supplementation strategy. Previous investigations revealed the efficacy of BCAAs supplementation with the pre-load strategy before resistance exercise [31,32]. Conversely, a beneficial effect of BCAAs supplementation emerged with a pre- and post-exercise strategy, without pre-loading [23]. Hence, different supplementation strategies might induce different results. However, the modalities of the exercise protocol executed with a different amount of eccentric muscle action may differentially induce muscle damage and soreness. Therefore, the effective dosage and timing of BCAAs supplementation should be considered in combination with the exercise modalities and intensities.

COD is an important process for team sport players [47] and the 505 COD test can assess rapid deceleration and re-acceleration phases [27,47] and potential performance impairment after an EIMD. Moreover, the test can isolate the COD deficit by considering the last portion of the deceleration and re-acceleration phase, where higher coordinative and technical demands are required to make faster COD [48]. The current results showed that the total completion time of the 505 COD test and COD deficit time only significantly increased post-exercise, indicating an impairment of COD ability after EIMD. However, for the COD deficit, the effect of supplementation highlighted a greater impairment under the placebo condition compared to the BCAAs condition. It seems that the isolation of the COD deficit was more sensitive to detect the potential beneficial effect of supplementation. The EIMD did not impair the neuromuscular performance at follow-up measurements (from 24 to 72 h). However, this is considered to only be initial evidence and more research is suggested to confirm this finding. First, the EIMD did not induce an impairment of muscle function at follow-up measurement, which is different from muscle damage and soreness markers. Hence, the association of muscle function with muscle damage and soreness should be clarified. Second, it could be speculated that this kind of test is too short to detect small performance changes several days after a damaging exercise protocol [30]. Further research is clearly required to clarify the association among several markers of EIMD and the sensitivity of short duration tests applied in the sport context.

The current study demonstrated a reduction of ankle-brachial index (ABI) after exercise, which may be related to the decrease in ankle pressure [49] and the falling of peripheral resistance [50] resulting in decreased pressure and increased blood perfusion in the working muscles [51]. Additionally, brachial–ankle pulse wave velocity (baPWV) has been acknowledged as an indicator of systemic arterial stiffness, whilst carotid–femoral PWV (cfPWV) represents the central arterial stiffness [52]. The intimate association between baPWV and cfPWV could indicate that baPWV might provide similar information derived from central arterial stiffness [28]. Contrary to the hypothesis of the exacerbation of arterial stiffness, an improvement in ABI and baPWV parameters after the exercise protocol was found and baPWV did not return to the baseline level until 48 h after exercise. Therefore, despite the elevation in muscle damage and inflammatory biomarkers, there was not a deterioration of vascular health after a COD sprinting exercise. Comparing different modalities of eccentric-based exercise, studies have demonstrated an increase in arterial stiffness after downhill running [53] and no changes after eccentric cycling [54], which is contrary to the results of the current study. Moreover, a previous meta-analysis comparing resistance and endurance exercise demonstrated a greater increase in arterial stiffness after resistance exercise [55]. It could be speculated that the results of the current study could be attributed to the fitness level of participants and the training adaptations induced by the nature of the sport (i.e., combination of aerobic and anaerobic endurance adaptations), ultimately leading to adaptive responses of the arteries and endothelium [56]. Moreover, the reduction of arterial stiffness might be associated with the autonomic nervous system activity, in particular the response of sympathetic nervous system. Generally, an increase in sympathetic nerve activity during exercise leads to the vasoconstriction in active muscle, which is essential to maintain stable blood pressure and cardiac output [57]. However, the vasoconstriction stimulated by the sympathetic nervous system could be reduced based on the exercise mode and shear stress patterns [58], and might be associated with the vasodilation which occurs from the sympathetic activation with the endothelium-mediated vasodilator effect overriding the neural vasoconstriction effect [58,59]. Taken together, arterial stiffness can be influenced by exercise modality, duration, and intensity, training status, and magnitude of muscle damage. Importantly, in an athletic population, muscle damage may not influence arterial stiffness.

This is the first study that investigated the potential effect of BCAAs supplementation on vascular health following repeated bouts of COD sprinting. Previous data supports the notion that EIMD may induce detrimental effects on arterial stiffness and local endothelial function [11,41]. In term of BCAAs supplementation, a previous study demonstrated that BCAAs could affect the releasing of pro-inflammatory cytokines, such as IL-6 [42], in the inflammatory response, which is associated with the increasing of endothelial dysfunction and arterial stiffness [60]. A recent study demonstrated that BCAAs supplementation could alter the immunomodulatory capacity of mesenchymal stem cells by promoting the regulation and immunological suppression, and reduce the expression of pro-inflammatory cytokines production by macrophages [61]. It could be speculated that the reduction in arterial stiffness from post-exercise until 48 h in the BCAAs condition might be associated with the beneficial effect of BCAAs in suppressing the pro-inflammatory cytokines during inflammatory responses. However, we failed to demonstrate the effectiveness of BCAAs supplementation on arterial stiffness due to the lack of worsening in arterial stiffness after the COD exercise protocol and significant differences between the two conditions did not emerge. Thus, the effect of BCAAs supplementation in mitigating the arterial stiffness after EIMD remains unclear and should be explored in the future.

Previous evidence suggests that muscle damage and soreness are related to arterial stiffness after exercise [41,62]. However, the current analysis only showed a negative large association between muscle soreness and arterial stiffness at 24 h after exercise. Considering this initial finding, it is not possible to derive strong conclusions and the comparison with previous evidence is limited since this was the first study investigating the magnitude of muscle damage induced by a 180° COD sprinting exercise.

This study has strengths that support the obtained results. The first is the considerable sample size, that is, 19 collegiate basketball players with experience in COD. Moreover, all participants were fully familiarized with the experimental procedures and the study was conducted using a randomized, crossover, double-blind, and placebo-controlled design. Finally, diet and exercise habits were standardized during the two trials. However, the current study had some limitations that should be considered in planning future studies. First, the supplementation strategy in terms of dosage (0.17g/kg BCAAs) and timing (30 min before exercise and post-exercise immediately) might not be sufficient to reduce the negative effects of EIMD in trained populations. Moreover, the dietary regimen of athletes was not fully controlled between the two trials, even though we strictly instructed them to maintain their regular dietary regimen for the entire duration of the study. Second, the deleterious effects of muscle damage on arterial stiffness in this study were only expressed by the changes in systemic arterial stiffness. Moreover, vascular health was only measured at one time-point in the day of the exercise protocol, without the possibility of detecting acute changes during the entire day. Hence, future research could be designed with more time-point measurements at the day of the exercise protocol to investigate the profile of acute responses. Third, the beneficial effect of BCAAs supplementation might have been influenced by the individual responses, considering the high variability in the muscle damage and inflammatory biomarkers. Finally, it might be difficult to detect small changes in the neuromuscular performance with a 505 COD test at follow-up measurements (24–72 h), due to the sensitivity of the test to detect small changes. Therefore, the appropriate testing to evaluate the changes of neuromuscular performance after COD exercise protocol should be considered in future studies.

## 5. Conclusions

Our study demonstrated that a 0.17 g/kg of BCAAs supplementation at pre- and post-exercise had no beneficial effect in attenuating muscle damage and soreness, performance decrement, and arterial stiffness in trained males after a COD sprinting exercise protocol. However, the magnitude of muscle damage after an eccentric exercise protocol, such as COD, might not be sufficient to induce a worsening in neuromuscular performance, whilst an improvement in arterial stiffness could even be reached. However, the perceived muscle soreness remains an important indicator of training adaptations, but it does not strongly reflect the underlying mechanisms during the time course of recovery. Thus, future studies are still required to elucidate the effect of other BCAAs supplementation strategies on the attenuation of the negative consequences of EIMD in team sport players.

## Figures and Tables

**Table 1:** Changes of vascular health variables for BCAAs and placebo supplementation conditions during the time course of recovery (mean ± SD).

Parameters	Condition	PRE	POST	24 h	48 h	72 h
ABI	Placebo BCAAs	1.06 ± 0.06 1.04 ± 0.05	0.95 ± 0.09 0.92 ± 0.05	1.06 ± 0.05 1.06 ± 0.07	1.06 ± 0.05 1.06 ± 0.06	1.07 ± 0.06 1.07 ± 0.06
baPWV (cm/s)	Placebo BCAAs	1064 ± 80 1098 ± 73	1023 ± 94 1049 ± 97	1049 ± 67 1052 ± 65	1050 ± 67 1047 ± 62	1061 ± 67 1068 ± 61
Ankle SBP (mmHg)	Placebo BCAAs	124 ± 11 127 ± 12	111 ± 15 113 ± 11	126 ± 12 126 ± 11	125 ± 11 124 ± 11	127 ± 11 128 ± 10
Brachial DBP (mmHg)	Placebo BCAAs	61 ± 6 65 ± 8	62 ± 8 64 ± 8	61 ± 5 61 ± 7	61 ± 6 61 ± 6	62 ± 6 62 ± 6
Brachial SBP (mmHg)	Placebo BCAAs	116 ± 6 121 ± 11	117 ± 10 121 ± 11	117 ± 8 117 ± 8	116 ± 8 116 ± 10	118 ± 8 118 ± 8

ABI = ankle-brachial index, ankle MAP = ankle mean arterial pressure ankle SBP = ankle systolic blood pressure, ankle DBP = ankle diastolic blood pressure, baPWV = brachial-ankle pulse wave velocity, brachial SBP = brachial systolic blood pressure, brachial DBP = brachial diastolic blood pressure, and brachial MAP = brachial mean arterial pressure.

**Table 2:** Correlation between changes in indirect markers of muscle damage and soreness and arterial stiffness variables at 24 h after COD exercise protocol.

	CK	VAS	baPWV	Ankle SBP	ABI
CK	1	0.694 ^1^	−0.409	0.202	−0.102
VAS	0.694 ^1^	1	−0.477 ^1^	0.012	−0.074

ABI = ankle-brachial index, ankle SBP = ankle systolic blood pressure, baPWV = brachial-ankle pulse wave velocity, CK = creatine kinase, VAS = Visual Analog Scale, ^1^ and Significant correlation (*p* < 0.05).

## Data Availability

No new data were created or analyzed in this study. Data sharing is not applicable to this article.
